# Novel Anion Exchange Membrane Based on Poly(Pentafluorostyrene) Substituted with Mercaptotetrazole Pendant Groups and Its Blend with Polybenzimidazole for Vanadium Redox Flow Battery Applications

**DOI:** 10.3390/polym12040915

**Published:** 2020-04-15

**Authors:** Hyeongrae Cho, Vladimir Atanasov, Henning M. Krieg, Jochen A. Kerres

**Affiliations:** 1 Institute of Chemical Process Engineering, University of Stuttgart, 70199 Stuttgart, Germany; vladimir.atanasov@icvt.uni-stuttgart.de; 2 Faculty of Natural Science, North-West University, Potchefstroom 2520, South Africa; henning.krieg@nwu.ac.za; 3 Forschungszentrum Jülich GmbH, Helmholtz Institute Erlangen-Nürnberg for Renewable Energy, 91058 Erlangen, Germany

**Keywords:** poly(pentafluorostyrene), mercaptotetrazole, vanadium redox flow battery, anion exchange membranes, blends

## Abstract

In order to evaluate the performance of the anion exchange membranes in a vanadium redox flow battery, a novel anion exchange polymer was synthesized via a three step process. Firstly, 1-(2-dimethylaminoethyl)-5-mercaptotetrazole was grafted onto poly(pentafluorostyrene) by nucleophilic F/S exchange. Secondly, the tertiary amino groups were quaternized by using iodomethane to provide anion exchange sites. Finally, the synthesized polymer was blended with polybenzimidazole to be applied in vanadium redox flow battery. The blend membranes exhibited better single cell battery performance in terms of efficiencies, open circuit voltage test and charge-discharge cycling test than that of a Nafion 212 membrane. The battery performance results of synthesized blend membranes suggest that those novel anion exchange membranes are promising candidates for vanadium redox flow batteries.

## 1. Introduction

Vanadium Redox Flow Batteries (VRFBs) are electrochemical energy storage systems producing electricity from chemical redox reactions. Significant contributions to the development of the VRFB technology have been made at the University of New South Wales by Maria Skyllas-Kazacos’s group [1]. VRFBs are among the most promising energy storage systems for renewable power generation, due to their high energy efficiency, flexible design, unlimited capacity and satisfactory safety [2]. 

As an important component of the VRFBs, an ion exchange membrane, separates the two electrolytes to prevent the cross-mixing of the positive and negative electrolytes, while allowing the transport of ion species such as H^+^ when a cation-exchange membrane (CEM) is used as the separator material, or HSO_4_^−^ or SO_4_^2−^ when the two electrolyte compartments are separated by an anion-exchange membrane (AEM) to maintain charge balance [3]. Nafion^®^ type membranes developed by DuPont have been widely employed as the CEMs for VRFBs due to their high ionic conductivity and superior chemical stability; however, it has been found that Nafion^®^ type membranes suffer from high vanadium ion cross-over, resulting in fast capacity fade when used in VRFBs [4,5].

In the last decade, interest in using AEMs in various applications, such as alkaline anion exchange membrane fuel cells [6], alkaline anion exchange membrane water electrolysis [7], ionic polymer membrane actuators [8], dye-sensitized solar cells [9], CO_2_ pumps [10] and redox flow batteries [11] has strongly increased. In a recent review paper, the number of journal articles published in the field of anion exchange membranes was analyzed using Web of Science (SCI-EXPANDED), showing a steady growth since 2007 [12]. The significant advantage of AEMs when applied in a VRFB is that their positively charged fixed ion groups (commonly quaternary ammonium) exclude positively charged, strongly oxidizing vanadium ions from the membrane interior due to the Donnan exclusion effect, leading to extremely low vanadium ion cross-over [11].

Additionally, modified poly(pentafluorostyrene) (PPFSt) was applied in proton exchange membrane fuel cells as sulfonated [13] or phosphonated [14] CEMs, exhibiting outstanding oxidative and thermal stabilities, which were better than those of their non-fluorinated styrene polymer analogs due to the higher bond strengths of the C–F bonds compared to those of C–H bonds [15]. The outstanding stability of PPFSt-based CEMs inspired us to find a route to prepare a PPFSt-based anion exchange polymer with the advantages of AEMs. Introducing spacer units between the polymer backbone and the side chains in an AEM was thought to lead to an enhanced ion-conductivity, which might be facilitated by an improved phase separation between the hydrophobic polymer backbone and the cation-carrying side chains [16]. Tetrazole tethered polymers showed an improved conductivity compared to that of tetramethylammonium-based quaternized poly(phenylene oxide) AEMs, which is thought to be caused by the long-range hydrogen bond network formation by the tetrazole moiety contributing to the ion conductivity [17]. 

In this study, we synthesized AEMs based on poly(pentafluorostyrene) (PPFSt) by a three-step process: (1) the grafting of 1-(2-dimethylaminoethyl)-5-mercaptotetrazole, (2) the methylation (quaternization) of the tertiary amino groups, and (3) the blending of the anion-exchange polymer with polybenzimidazole. Due to the inability to prepare a free-standing membrane from the functionalized polymer, it was blended in the third step with the partially fluorinated and highly stable polybenzimidazole (F6-PBI) for enhancing the film-forming properties and mechanical strength of the AEMs [18,19]. In our earlier study, it has already been shown that polybenzimidazoles, as blend components, have a positive impact on the mechanically stability and toughness of the cation-exchange redox-flow battery separator blend membranes [20]. The properties of the blend membranes such as conductivity, thermal stability, dimensional stability and VRFB performance are described. The synthesized blend membranes in this study showed better VRFB performances than that of the Nafion membrane. To the best of our knowledge, AEMs based on PPFSt have not yet been reported.

## 2. Materials and Methods 

### 2.1. Materials

PPFSt was prepared as described in our earlier study [13]. F6-PBI was purchased from Yanjin Technology, Shenzhen, China. Sulfuric acid, methyl ethyl ketone, potassium hydroxide, 0.1 N standard hydrochloric acid and sodium chloride were purchased from Carl Roth GmbH, Karlsruhe, Germany. *N*-methyl-2-pyrrolidone (NMP) and diethyl ether were obtained from Sigma-Aldrich, Munich, Germany. *N*,*N*-dimethylacetamide was purchased from Acros Organics, Darmstadt, Germany. Sulfuric acid was purchased from VWR International, Bruchsal, Germany. 1-(2-dimethylaminoethyl)-5-mercaptotetrazole (DMAEMTZ) and triethylamine were purchased from TCI Chemicals, Eschborn, Germany. Iodomethane was purchased from Merck, Darmstadt, Germany. The vanadium electrolyte solution (1.6 M vanadium in 30% sulfuric acid: 50% VO^2+^ and 50% V^3+^) was supplied by RIVA GmbH Batteries, Backnang, Germany. Nafion^®^212 (Nafion) was purchased from Ion Power GmbH, Munich, Germany.

### 2.2. PPFSt Synthesis

PPFSt was obtained via the emulsion polymerization of pentafluorostyrene as described in the literature and as mentioned in the materials section (M_n_: 52 kDa, M_w_: 124 KDa, PDI: 2.4—determined by GPC in THF at 30 °C with standard polystyrene) [13].

^19^F NMR (400MHz, THF-d_8_, δ): −144 (t, 2F), −157 (s, 1F), −163 (s, 2F).

### 2.3. 1-(2-Dimethylaminoethyl)-5-Mercaptotetrazole Grafting onto PPFSt (PPFSt-MTZ)

A flask, which was equipped with a magnetic stirrer, a condenser, and an argon inlet and outlet, was charged with PPFSt (1 g, 5.152 mmol) in 40 mL of methyl ethyl ketone (MEK). After the complete dissolution of PPFSt, 20 mL of dry NMP were added. Subsequently, triethylamine (7.82 g, 77.28 mmol) and 1-(2-dimethylaminoethyl)-5-mercaptotetrazole (4.55 g, 25.76 mmol) were added. The mixture was stirred at 95 °C for 3 days. After cooling to room temperature, the reaction solution was dropwise precipitated in water. The polymer precipitate was filtered off and washed several times using an excess of deionized water. The final polymer was dried overnight in vacuum at 60 °C and 1.21 g of product was obtained. 

^1^H NMR (400 MHz, THF-d_8_, δ): 4.43 (s, 2H), 2.61 (s, 2H), 2.16 (s, 7H). 

^19^F NMR (400 MHz, THF-d_8_, δ): −134 (s), −142 (s), −156 (s), −163 (s).

### 2.4. Quaternization of PPFSt-MTZ (M-PPFSt-MTZ)

For the quaterization of PPFSt-MTZ, 0.5 g of PPFSt-MTZ was dissolved in 20 mL of NMP. A high excess of iodomethane (0.89 mL, 2000% excess) was added to the reaction medium using a syringe and stirred at room temperature for 4 days to achieve full conversion. Subsequently, the reaction solution was added dropwise to diethylether, where the polymer precipitated. The precipitate was filtered and washed twice with diethylether and dried overnight under vacuum at 60 °C. 0.56 g of a yellowish powder was obtained. 

^1^H NMR (400MHz, DMSO-d_6_, δ): 5.06 (s, 2H), 4.08 (s, 2H), 3.28 (s, 8.5H + water).

### 2.5. Membrane Preparation

F6-PBI was dissolved at 80 °C in NMP as a 5 wt. % solution. The M-PPFSt-MTZ was dissolved in NMP, also as a 5 wt. % solution. After the complete dissolution of the polymers in the solvent, the two polymer solutions were mixed at room temperature. Subsequently, the mixed solution was cast into a petri-dish or onto a glass plate and placed in an oven at 80 °C for 16 h to evaporate the solvent. The dried membrane was peeled off from the glass supports by immersion in deionized water at room temperature. The resulting membrane was soaked in 1 M sulfuric acid for 24 h, which was replaced once with fresh sulfuric acid during this period, before immersing the membranes in DI water for 24 h to remove excess sulfuric acid from the membrane surfaces.

### 2.6. Ion Exchange Capacity (IEC)

IEC was measured as described in the literature [18]. IEC was calculated by the back-titration method. Accordingly, the membrane sample was immersed in a 1 M KOH solution at 90 °C for 1 day to allow full ion exchange to occur. After intense washing with DI water, the membrane was immersed in a saturated sodium chloride solution for 1 day. Then, 3 mL of standard 0.1 N hydrochloric acid was added, and the mixture was stirred for 1 day. The resulting solution was titrated with standard 0.1 N sodium hydroxide solution. The membrane was, again, washed intensely with deionized water and dried overnight at 90 °C. The dry weight of the membrane was then determined. The IEC was calculated using the following equation (Equation (1)).

(1) $$\mathrm{IEC}=\frac{C_{\mathrm{HCl}}\times V_{\mathrm{HCl}}-C_{\mathrm{NaOH}}\times V_{\mathrm{NaOH}}}{m_{\mathrm{dry}}}$$

where IEC is the ion exchange capacity (OH form, mmol/g), *C*_HCl_ is the concentration of the hydrochloric acid solution (mmol/mL), *V*_HCl_ is the volume of the hydrochloric acid solution used (mL), *C*_NaOH_ is the concentration of the sodium hydroxide solution (mmol/mL), *V*_NaOH_ is the added volume of the sodium hydroxide solution (mL), and *m*_dry_ is the dry weight of the membrane (g).

### 2.7. Conductivity

Membrane resistances were measured by electrochemical impedance spectroscopy using a Zahner elektrik IM6 device (Zahner-elektrik GmbH, Kronach, Germany) in 1 M sulfuric acid at room temperature. The resistance was investigated in a frequency range of 200 KHz–8 MHz by intercepting the impedance with the real *x*-axis. Four samples per membrane were measured. The conductivity was calculated using the following equation (Equation (2)).

(2) $$\sigma =\frac{1}{R_{\mathrm{sp}}}=\frac{d}{R\times A}$$

where σ is the conductivity (S/cm), *R*_sp_ is the resistivity (Ω cm), *d* is the thickness of membrane (cm), *R* is the ohmic resistance (Ω), and *A* is the electrode area (cm^2^).

### 2.8. Swelling Ratio (%) and Water Uptake (%)

The swelling ratio was characterized by the dimensional expansions of length, width and thickness (SRL, SRW and SRT respectively), which was measured by determining the difference between the wet and dry states of the membrane (Equations (3)–(5)). Similarly, the relative water uptake (WU) was determined by determining the weight difference between the wet and dry membrane (Equation (6)). In each case, five membrane samples were used, from which an average was obtained.

(3) $$\mathrm{SRL}\;\left(\%\right)=\frac{\left(\mathrm{Wet}\;\mathrm{length}\;-\;\mathrm{Dry}\;\mathrm{length}\right))}{\mathrm{Dry}\;\mathrm{length}}\times 100$$

(4) $$\mathrm{SRW}\;\left(\%\right)=\frac{\left(\mathrm{Wet}\;\mathrm{width}\;-\;\mathrm{Dry}\;\mathrm{width}\right)}{\mathrm{Dry}\;\mathrm{width}}\times 100$$

(5) $$\mathrm{SRT}\;\left(\%\right)=\frac{\left(\mathrm{Wet}\;\mathrm{thickness}\;-\;\mathrm{Dry}\;\mathrm{thickness}\right)}{\mathrm{Dry}\;\mathrm{thickness}\;}\times 100$$

(6) $$\mathrm{WU}\;\left(\%\right)=\frac{\left(\mathrm{Wet}\;\mathrm{weight}\;-\;\mathrm{Dry}\;\mathrm{weight}\right)}{\mathrm{Dry}\;\mathrm{weight}}\times 100$$

### 2.9. Thermal Stability 

The thermal stability of the polymers was investigated using a Netzsch STA 449 F3 (Netzsch, Selb, Germany). The temperature was increased, with a heating rate of 20 °C/min, from 30 °C to 600 °C, under an O_2_/N_2_ atmosphere (O_2_: 56 mL/min, N_2_: 24 mL/min). Decomposed gases coming out during the thermal gravimetric analysis (TGA) were analyzed by FT-IR coupled to TGA. 

### 2.10. Vanadium Redox Flow Battery Performance

To evaluate the vanadium redox flow battery performance, a single cell was built as described in the literature [21]. The membrane was placed between two electrodes (active area: 28 cm^2^, SIGRACELL^®^ GDF 4.6 EA, SGL Carbon GmbH, Bonn, Germany,) which were thermally treated at 450 °C for 5 h after immersion in 1 M sulfuric acid for 1 day. Copper plates were used for loading and as current collectors. The cell was assembled with screws by applying a torque of 3.5 Nm. Twenty milliliters of a vanadium electrolyte (1.6 M vanadium in 30% sulfuric acid: 50% VO^2+^ and 50% V^3+^) was used on both the anode and cathode sides. All the measurements were performed in a stationary mode, i.e., without electrolytes flowing through the cell. The cell was initially charged to 1.6 V and discharged to 1.0 V. The cell was charged with 40 mA/cm^2^ and discharged with different current densities to obtain the coulombic efficiency (CE), voltage efficiency (VE) and energy efficiency (EE). The open circuit voltage (OCV, self-discharge test) was monitored as a function of time after charging the cell to 1.6 V. A long term charging-discharging cycling test was performed, with a current density of 40 mA/cm^2^ for both charging and discharging. All experiments of the vanadium redox flow battery were conducted under ambient temperature. The CE, VE and EE were calculated as follows.

(7) $$\mathrm{CE}\;\left(\%\right)=\frac{\mathrm{discharge}\;\mathrm{capacity}}{\mathrm{Charge}\;\mathrm{capacity}}\times 100$$

(8) $$\mathrm{VE}\;\left(\%\right)=\frac{\mathrm{average}\;\mathrm{discharge}\;\mathrm{capacity}}{\mathrm{average}\;\mathrm{charge}\;\mathrm{capacity}}\times 100$$

(9) EE (%)=CE ×VE

## 3. Results and Discussion

### 3.1. Synthesis of M-PPFSt-MTZ

**Figure 1:**
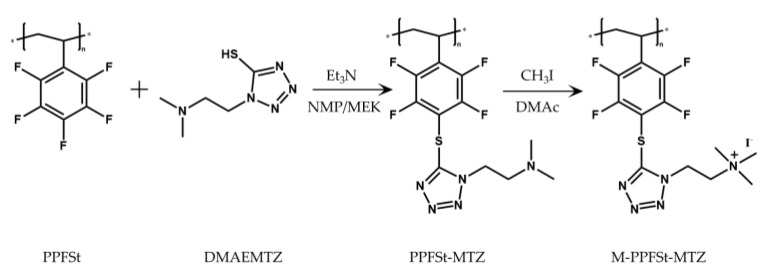
Anion exchange polymer preparation.

**Figure 2:**
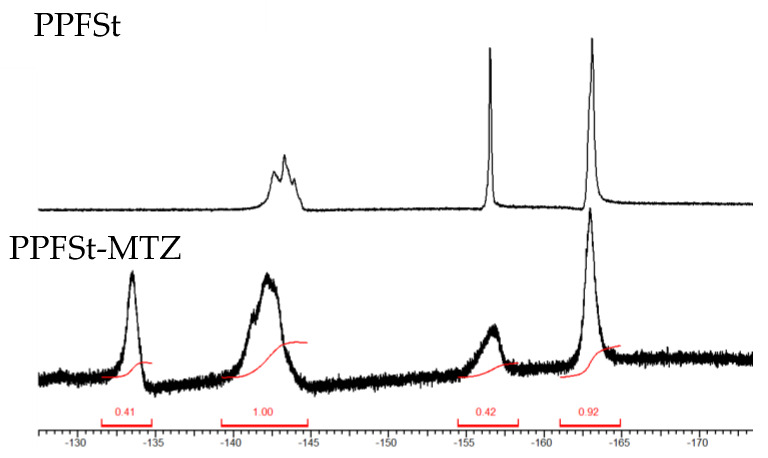
The ^19^F-NMR spectra of poly(pentafluorostyrene) (PPFSt) and PPFSt-MTZ.

The M-PPFSt-MTZ was synthesized according to the reaction scheme described in Figure 1. Firstly, the grafting of 1-(2-dimethylaminoethyl)-5-mercaptotetrazole onto PPFSt was conducted according to the literature, where mercaptoalcohols were grafted onto the PPFSt by the nucleophilic displacement of the 4-F of the pentafluorophenyl moiety with the thiolate [22]. According to the literature, when using MEK as a solvent for the reaction of PPFSt with mercaptoalcohols, an almost 100% degree of substitution (DOS) was achieved at 50 °C after 50 h. Therefore, PPFSt-MTZ was initially synthesized in MEK. However, when 1-(2-dimethylaminoethyl)-5-mercaptotetrazole was added to the reaction solution, it did not fully dissolve. Thus, an additional 20 mL of dry NMP was added to the reaction vessel, resulting in complete dissolution. Then, the reaction mixture was heated to 95 °C for 3 days (see Table S1 in the supporting information for the optimization of the reaction). As shown in Figure 2, the ^19^F NMR spectra of the polymers were recorded. The new peak at −134 ppm on the ^19^F NMR spectra, that can be attributed to the grafting of the 1-(2-dimethylaminoethyl)-5-mercaptotetrazole, was used to calculate the DOS according to the literature [22]. For this specific synthesis, the calculated DOS was 41%. For the DOS calculation, see Figure S1 of the supporting information. 

**Figure 3:**
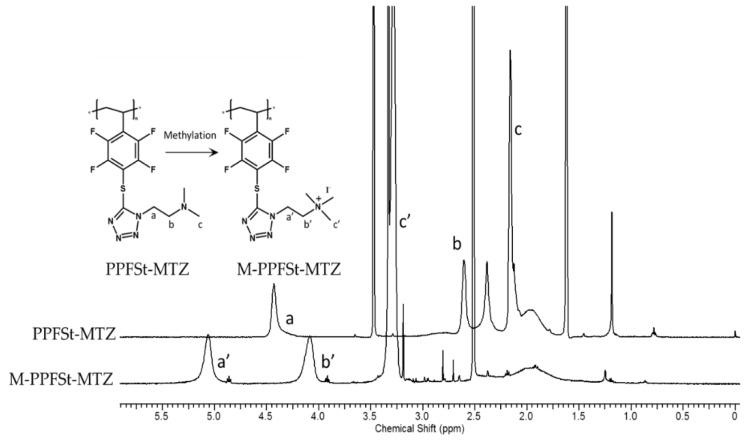
The ^1^H-NMR spectra of PPFSt-MTZ and M-PPFSt-MTZ.

For the quaternization of PPFSt-MTZ, the tertiary amine groups were methylated using iodomethane (see Figure 1). The methylation of tertiary amines with a large excess of iodomethane for complete quaternization has been widely used for anion exchange membrane preparation [23,24,25,26]. The ^1^H NMR spectra of both PPFSt-MTZ and M-PPFSt-MTZ are shown in Figure 3. The CH_3_ signal of PPFSt-MTZ on nitrogen was observed at 2.16 ppm. After methylation, the CH_3_ signal had shifted completely to 3.28 ppm due to the de-shielding from the quaternized nitrogen, indicating complete quaternization by iodomethane.

### 3.2. Membrane Preparation and Characterization

The details of the preparation and the properties of the blend membranes are presented in Table 1. Firstly, the blend membranes were prepared by using 90 wt. % and 80 wt. % of M-PPFSt-MTZ (M-PPFSt-MTZ-Me 1 and M-PPFSt-MTZ-Me 2, respectively), and 10 wt. % and 20 wt. % of F6-PBI, respectively. However, the membranes were found to be mechanically unstable after solvent evaporation and broke into pieces (see Figure S2 and Table S2 in the supporting information), probably due to the strong intermolecular hydrogen bonding and dipole-dipole association of the highly functionalized with charged functional groups of PPFSt. In the literature, a similar polymer bearing tetrazole as a functional group has been reported to form mechanically stable membranes at a low functionalization degree [17]. Therefore, it is thought that the mechanical stability of membranes can be improved by decreasing the degree of functionalization or increasing the molecular weight of the PPFSt [27,28]. This issue will be addressed in future work. Another approach to improving mechanical stability entails the addition of a polymer that possesses good film-forming properties and stability. Thus, blend membranes were prepared by blending M-PPFSt-MTZ with polybenzimidazole (F6-PBI) containing 70 wt. % and 60 wt. % of M-PPFSt-MTZ in the blend membrane (M-PPFSt-MTZ-Me 3 and M-PPFSt-MTZ-Me 4, respectively; see Table 1). This gave us access to mechanically stable blend membranes based on the newly obtained M-PPFSt-MTZ.

**Figure 4:**
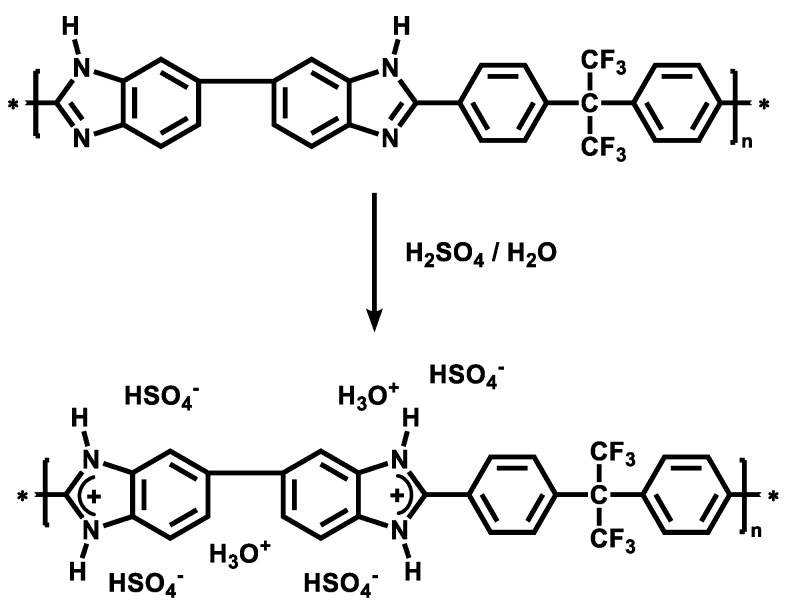
The protonation of partially fluorinated and highly stable polybenzimidazole (F6-PBI) when doped with sulfuric acid [31].

The IECs of the blend membranes were relatively low (up to 1.6 mmol/g) as compared to other published results, due to the low DOS [29,30]. Accordingly, the blend membranes showed low chloride conductivities of 0.043 and 0.038 mS/cm for M-PPFSt-Me 3 and M-PPFSt-Me 4, respectively, measured in 1 M sodium chloride solution. However, when the anion exchange membranes were acidified using sulfuric acid or phosphoric acid, they absorbed more sulfate or bisulfate ions per anion exchange group via hydrogen bridges, resulting in higher conductivities than in their pure form. In 1 M sulfuric acid, conductivities of 19.1 ± 1.64 mS/cm for M-PPFSt-MTZ-Me 3 and 13.5 ± 0.68 mS/cm for M-PPFSt-MTZ-Me 4 were measured. It is known, as shown in Figure 4, that the imidazole moiety of PBI is protonated in an acidic medium, with hydrogen sulfate counter ions significantly contributing to ion conductivity [31]. Thus, the conductivity of pure F6-PBI membrane was also investigated, which was 6.22 ± 0.27 mS/cm in 1 M sulfuric acid. It is thus clear that the conductivity was increased by blending compared to the conductivity of the pure F6-PBI membrane, indicating that the increase in conductivity originated from the anion exchange groups of the novel anion-exchange polymer synthesized in this study. The water uptake of membranes is one of the important parameters, as a higher water uptake of membranes normally results in excessive swelling, leading to poor dimensional stability. The water uptake of the M-PPFSt-MTZ-Me 3 membrane was higher than that of the M-PPFSt-MTZ-Me 4 membrane due to the somewhat higher IEC. The blend membranes in this study showed dimensional stabilities comparable to values in the literature, being lower than 10% [32].

**Figure 5:**
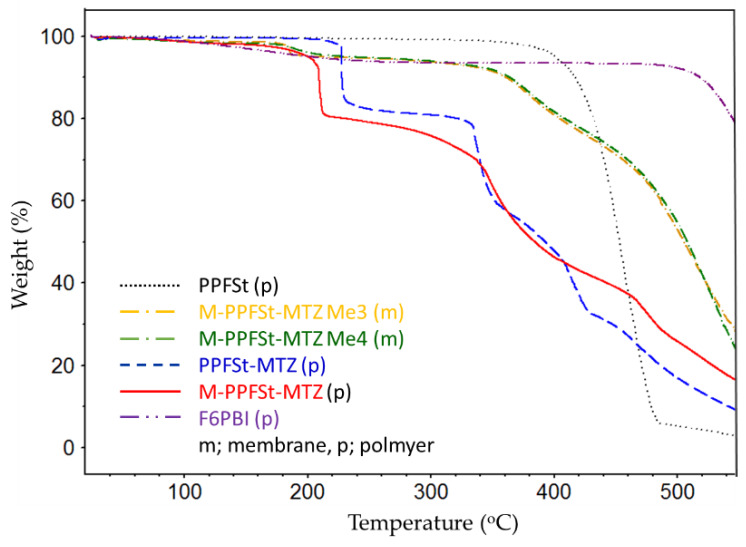
The thermal stabilities of polymers and membranes used in this study.

In order to investigate the thermal stability of the synthesized polymer and membranes, the weight loss was determined as a function of temperature using thermal gravimetric analysis (TGA). The TGA was operated with a highly oxidative atmosphere (70% oxygen, 30% nitrogen). According to Figure 5, PPFSt had a thermal resistance remaining stable up to 340 °C. After modification of the polymer (PPFSt-MTZ and M-PPFSt-MTZ), its thermal stability decreased. The first weight loss step at around 150 °C for M-PPFSt-MTZ can be attributed to water evaporation. For PPFSt-MTZ, the second weight loss step starts at 2 °C and can be ascribed to the loss of side chains due to the instability of the mercaptotetrazole rings (see Figure S3a in the supporting information). After methylation, the second weight loss above 189 °C can be assigned to the loss of iodomethane via nucleophilic attack and mercaptotetrazole side chains (see Figure S3a in the supporting information). The temperature of polymer main chain degradation was indicated to be around 340 °C (see Figure S3b in the supporting information). However, in spite of the decline in stability after modification, the synthesized anion exchange polymer and blend membranes showed sufficient thermal stability, remaining stable up to 189 °C under harsh oxidative conditions.

### 3.3. Vanadium Redox Flow Battery Evaluation 

**Figure 6:**
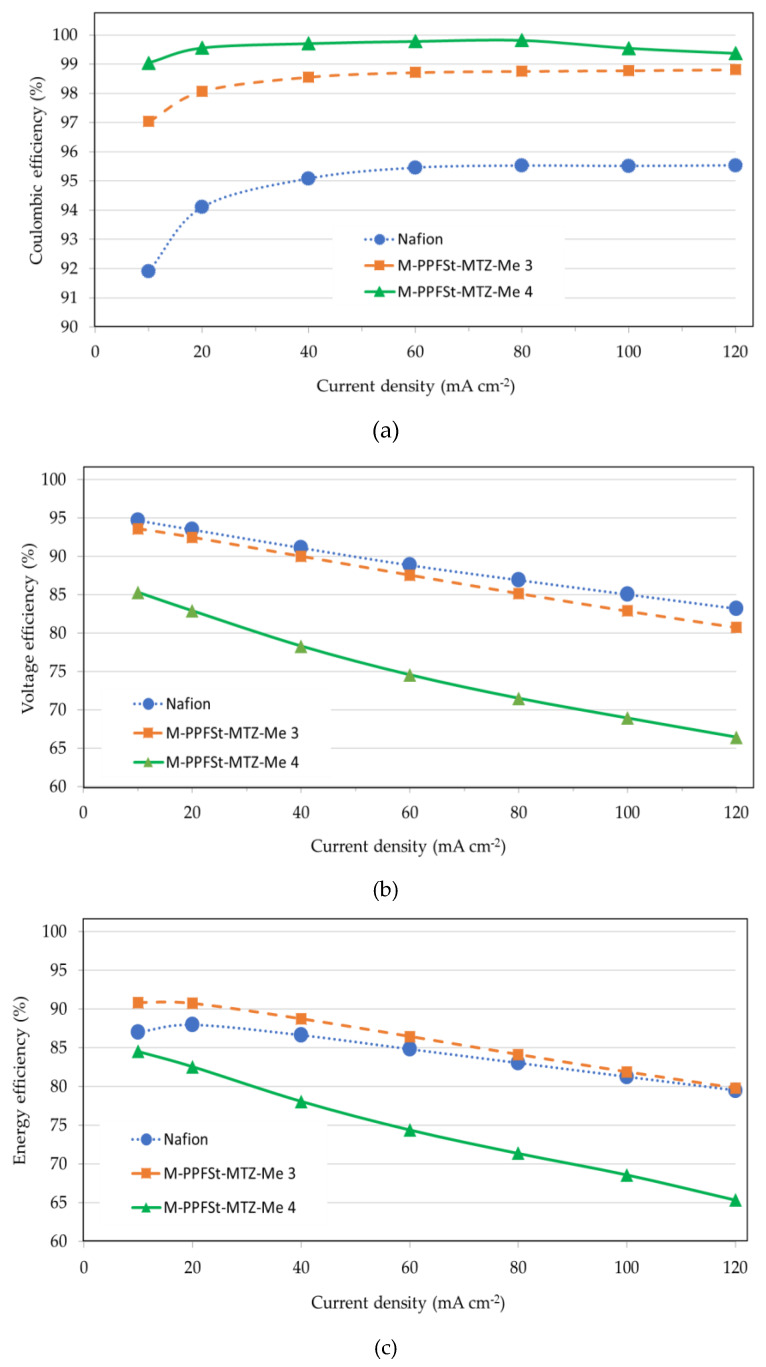
The coulombic efficiency (**a**), voltage efficiency (**b**) and energy efficiency (**c**) of Vanadium Redox Flow Batteries (VRFBs) as a function of the current density.

In order to evaluate the VRFB performance, a single VRFB cell was constructed as described in the literature [21]. It is important to note that all of the measurements were performed without electrolyte circulation, in a stationary mode. This allowed for a quick equilibration of the cell due to the relatively low electrolyte volume used (20 mL on each side). Thus, the coulombic efficiencies (CEs) were investigated at various current densities. CE is determined by the ratio of charging time to discharging time. CE is influenced by the cross-over of vanadium ions through the membrane and increases with increasing current density due to the faster cross-over of vanadium ions [33]. As seen in Figure 6a, the CEs of blend membranes were higher than that of the Nafion membrane, due to the Donnan exclusion effect as described above, while the Nafion membrane showed a low CE due to increased vanadium cross-over. It should be noted that AEMs do not always show high CEs, as this would also require a high dimensional stability of the membrane [21]. In addition, more dense PBI membranes yielded higher CEs due to the low vanadium ion permeability [34]. Accordingly, the M-PPFSt-MTZ-Me 4 membrane with the highest F6-PBI content also showed the highest CE, of almost 100%. 

Voltage efficiencies (VEs) are governed by the ohmic resistances between two electrodes, which are mainly affected by the membrane resistance, corresponding to its ionic conductivity [35]. As shown in Figure 6b, the Nafion membrane showed the highest VE irrespective of the current density, correlating with its highest conductivity among the membranes tested in this study. On the other hand, the M-PPFSt-MTZ-Me 4 showed the lowest VE, in line with its lowest conductivity among all of the membranes tested in this study.

The overall energy efficiencies (EEs) of the VRFBs are defined as the CEs multiplied by the VEs, of which the results are shown in Figure 6c. The EE of M-PPFSt-MTZ-Me 3 showed slightly higher values than that of Nafion212 irrespective of the current density. This blend composition for the membrane (60% M-PPFSt-MTZ and 40% F6-PBI) seemed to provide the best compromise between vanadium cross-over and membrane conductivity. On the other hand, the EE of M-PPFSt-MTZ-Me 4 was the lowest for all measured current densities due to the low VE in spite of the high CE.

**Figure 7:**
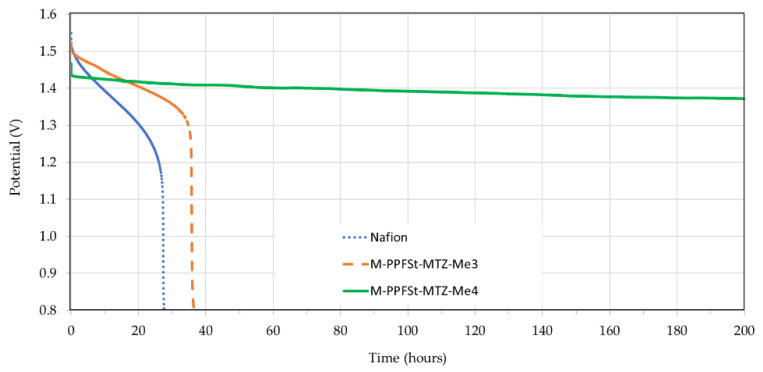
Open circuit voltage (OCV) of VRFBs with Nafion and blend membranes as a function of time.

Open circuit voltage (OCV) measurements can be used to indirectly measure the vanadium ion cross-over [36], as the cross-over through the membrane results in self-discharge, which corresponds to a OCV drop in the performance of the VRFB [37]. Thus, self-discharge was monitored as a function of time. As seen in Figure 7, the OCV when using M-PPFSt-MTZ-Me 4 membranes remained nearly stable for over 200 h, indicating a low vanadium ion cross-over, as expected. When using Nafion212 in the VRFB, a fast self-discharge time of 27 h was observed, due to fast cross-over. A VRFB with the M-PPFSt-MTZ-Me 3 membrane showed a slightly longer self-discharge time of 35 h, compared to that with Nafion. It is clear that the results of the OCV measurements are in accordance with the results obtained from the CE test.

**Figure 8:**
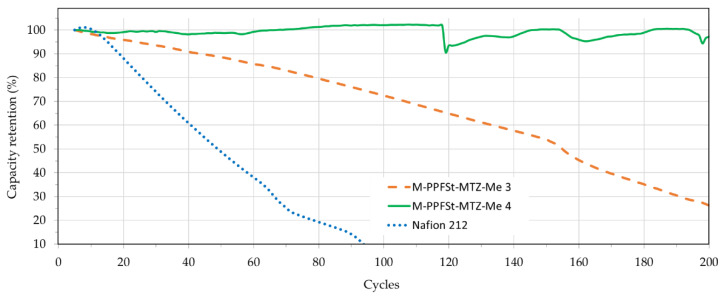
The charging-discharging cycling test.

Charging-discharging cycling performance, with operation at a constant current density of 40 mA/cm^2^ for both the charging and discharging of the blend membranes, was measured and compared with Nafion212 (Figure 8). It must be remembered that some capacity decay is inevitable during cycling tests, due to the different permeabilities of the four vanadium ion states, as well as the water transfer between electrolytes through the membrane [38,39]. While this capacity decay can be restored by simply remixing the two electrolytes [40], this will add to the cost of maintaining the VRFBs. Thus, capacity retention is one of the most critical parameters in determining the success of a VRFB’s application. As seen in Figure 8, the VRFB with Nafion showed a fast decay in capacity, with most of its capacity lost in less than 100 cycles. By contrast, the VRFB with the M-PPFSt-MTZ-Me 3 membrane showed a much better capacity retention, with an 80% loss over 200 cycles, confirming considerably less vanadium ion cross-over. As in the previous experiment (CE and OCV), the VRFB with M-PPFSt-MTZ-Me 4 showed the highest capacity retention, again confirming both its chemical and mechanical stability and consistently low vanadium ion cross-over rate.

## 4. Conclusions

In this study, a novel anion exchange polymer based on quaternary ammonium-functionalized poly(pentafluorostyrene) (PPFSt) was synthesized in two steps: (1) the grafting of 1-(2-dimethylaminoethyl)-5-mercaptotetrazole by nucleophilic F/thiolate exchange and (2) quaternizing the 2-dimethylaminoethyl group using iodomethane. This polymer showed sufficient thermal stability for the VRFB application. However, no mechanically stable free-standing membranes could be prepared from this polymer. Therefore, the anion-exchange polymer was blended with F6-PBI. The blend membranes were characterized and applied in vanadium redox flow batteries. Specifically, the blend membranes containing 30% and 40% of F6-PBI demonstrated suitable membrane properties in terms of IEC, conductivity and degree of swelling. During the VRFB test, the blend membrane (M-PPFSt-MTZ-Me 3) yielded a better performance in VRFBs in terms of energy efficiency, OCV and charging-discharging cycling than a Nafion membrane, while the blend membrane M-PPFSt-MTZ-Me 4 displayed the highest OCV and capacity retention of all of the membranes tested. It can therefore be concluded that these types of blend membrane are promising candidates for vanadium redox flow battery applications.

## Figures and Tables

**Table 1:** The properties of blend membranes and Nafion212.

Membranes	M-PPFSt-MTZ/ F6-PBI (by Weight)	IEC (mmol/g)	Conductivity (mS/cm)	Water Uptake (%)	Dimensional Stability (%)		
					Length	Width	Thickness
M-PPFSt-MTZ-Me 1^-^	9/1	-	-	-	-	-	-
M-PPFSt-MTZ-Me 2^-^	8/2	-	-	-	-	-	-
M-PPFSt-MTZ-Me 3	7/3	1.60	19.1 ± 1.64	12 ± 1.9	8 ± 0.6	5 ± 0.7	4 ± 1.2
M-PPFSt-MTZ-Me 4	6/4	1.15	13.5 ± 0.68	10 ± 2.2	7 ± 0.7	7 ± 0.7	4 ± 1.2
N 212	n.a	0.88	98.5 ± 4.95	13 ± 1.2	11 ± 0.6	8 ± 1.5	13 ± 2.2

- Membranes mechanically unstable after solvent evaporation; ^n.a^ not applicable.

## Supplementary Materials

The following are available online at https://www.mdpi.com/2073-4360/12/4/915/s1, Table S1: Optimization for PPFSt-MTZ synthesis, Figure S1: Calculation of degree of substitution confirming 41%, Figure S2: Photographs of the blend membranes, Table S2: The blend membranes preparation, Figure S3: FT-IR spectrum of decomposed gases from first degradation step (a) and polymer main chain degradation (b).
