# Supplementary material for: Novel Anion Exchange Membrane Based on Poly(Pentafluorostyrene) Substituted with Mercaptotetrazole Pendant Groups and Its Blend with Polybenzimidazole for Vanadium Redox Flow Battery Applications
Source: Polymers (Basel). 2020 Apr 15;12(4):915. doi: 10.3390/polym12040915 (PMC7240731; doi:10.3390/polym12040915)
Supplement: Supplementary file 1 [file polymers-12-00915-s001.pdf]

**Novel anion exchange membrane based on poly(pentafluorostyrene) substituted with mercaptotetrazole pendant groups and its blend with polybenzimidazole for vanadium redox flow battery application**

Hyeongrae Cho,[1] Vladimir Atanasov,[1] Henning M Krieg,[2] Jochen A Kerres[1,2,3]

[1]Institute of Chemical Process Engineering, University of Stuttgart, 70199 Stuttgart, Germany

[2] Faculty of Natural Science, North-West University, Focus Area: Chemical Resource Beneficiation, Potchefstroom 2520, South Africa

[3] Forschungszentrum Jülich GmbH, Helmholtz Institute Erlangen-Nürnberg for Renewable Energy, 91058 Erlangen, Germany

Correspondence: *hyeongrae.cho@icvt.uni-stuttgart.de; j.kerres@fz-juelich.de*

Table S1. Optimization for PPFSt-MTZ synthesis.

| Entry | Solvent | Equivalents of 1-(2-Dimethylaminoethyl)-5-mercaptotetrazole | Reaction conditions | Substitution Results* |
|---|---|---|---|---|
| 1 | NMP / MEK | 5 | 80 °C, 18 hours | Low substitution (1% DOS) |
| 2 | MEK | 1 | 55 °C, 72 hours | No substitution |
| 3 | NMP / MEK | 5 | 80 °C, 72 hours | Low substitution (15% DOS) |
| 4 | NMP / MEK | 5 | 95 °C, 72 hours | Used in this study (41% DOS) |
| 5 | NMP / MEK | 5 | 95 °C, 168 hours | 40 % DOS |

* Degree of substitution (DOS) was calculated by F-NMR

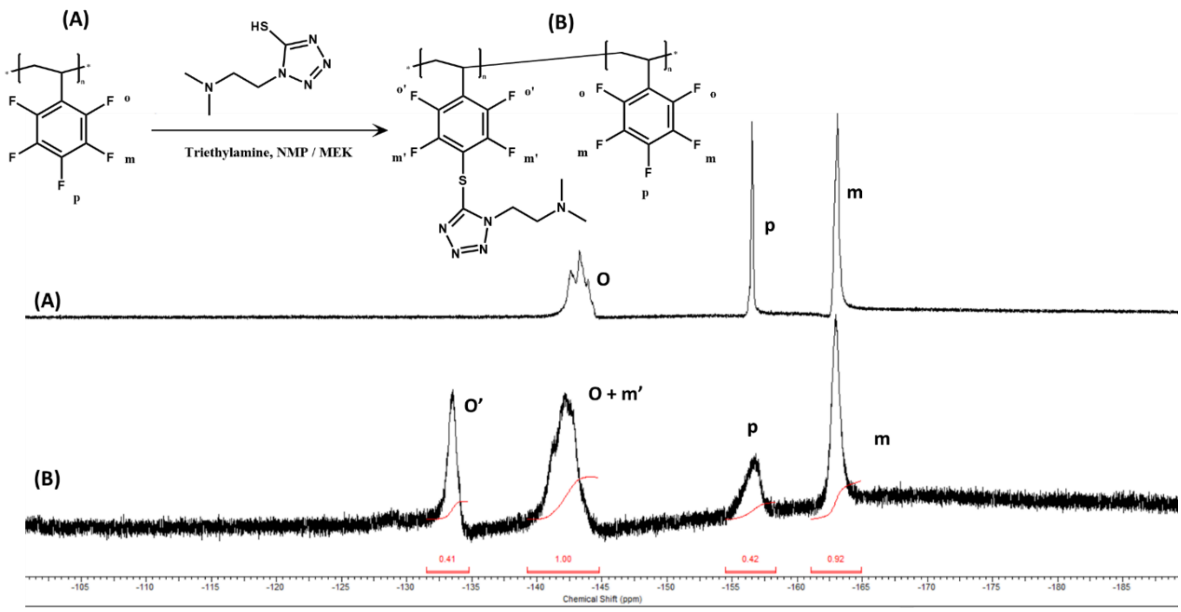


Figure S1. Calculation of degree of substitution confirming 41%.

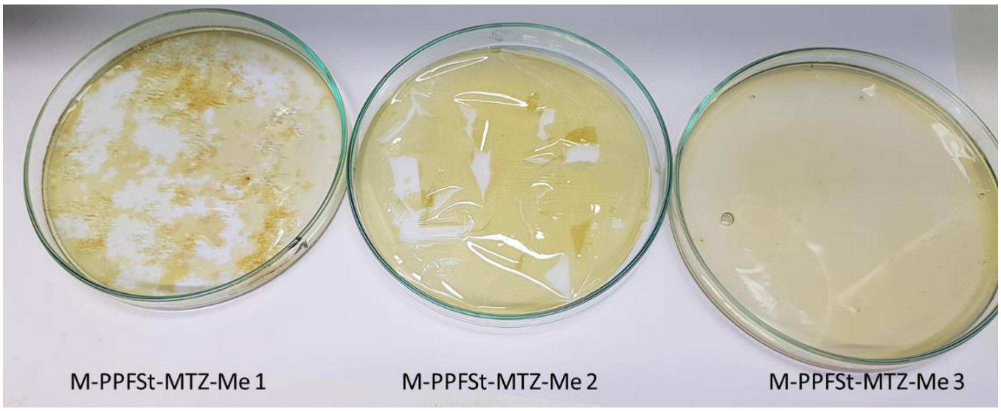


Figure S2. Photographs of the blend membranes.

Table S2. The blend membranes preparation.

| Membrane | M-PPFSt-MTZ : F6-PBI (by weight) | Membrane formation (see Figure S2) |
|---|---|---|
| M-PPFSt-MTZ-Me 1 | 9 : 1 | Brittle |
| M-PPFSt-MTZ-Me 2 | 8 : 2 | Brittle |
| M-PPFSt-MTZ-Me 3 | 7 : 3 | Ductile |
| M-PPFSt-MTZ-Me 4 | 6 : 4 | Ductile |

The blend membrane of M-PPFSt-MTZ-Me 3 was mechanically stable after drying the

solvent. Therefore, blend membranes of M-PPFSt-MTZ-Me 3 and M-PPFSt-MTZ-Me 4 were prepared as larger membranes onto glass plate for the investigation in this study.

As seen in the Figure S3 (a), a strong peak 2957 $cm^{-1}$ can be assigned to C-H vibration of N-$CH_3$ from the 1-(2-dimethylaminoethyl)-5-mercaptotetrazole. A strong peak at 1264 $cm^{-1}$ can be assigned to the iodomethane by a nucleophilic substitution of iodide. The peaks at 2200 and 2400 $cm^{-1}$ can be assigned to the loss of side chains,1-(2-dimethylaminoethyl)-5-mercaptotetrazole. Above 340°C, polymer main back bone degradation was observed for all polymers, PPFSt, PPFSt-MTZ and M-PPFSt-MTZ (Figure S3 (b).

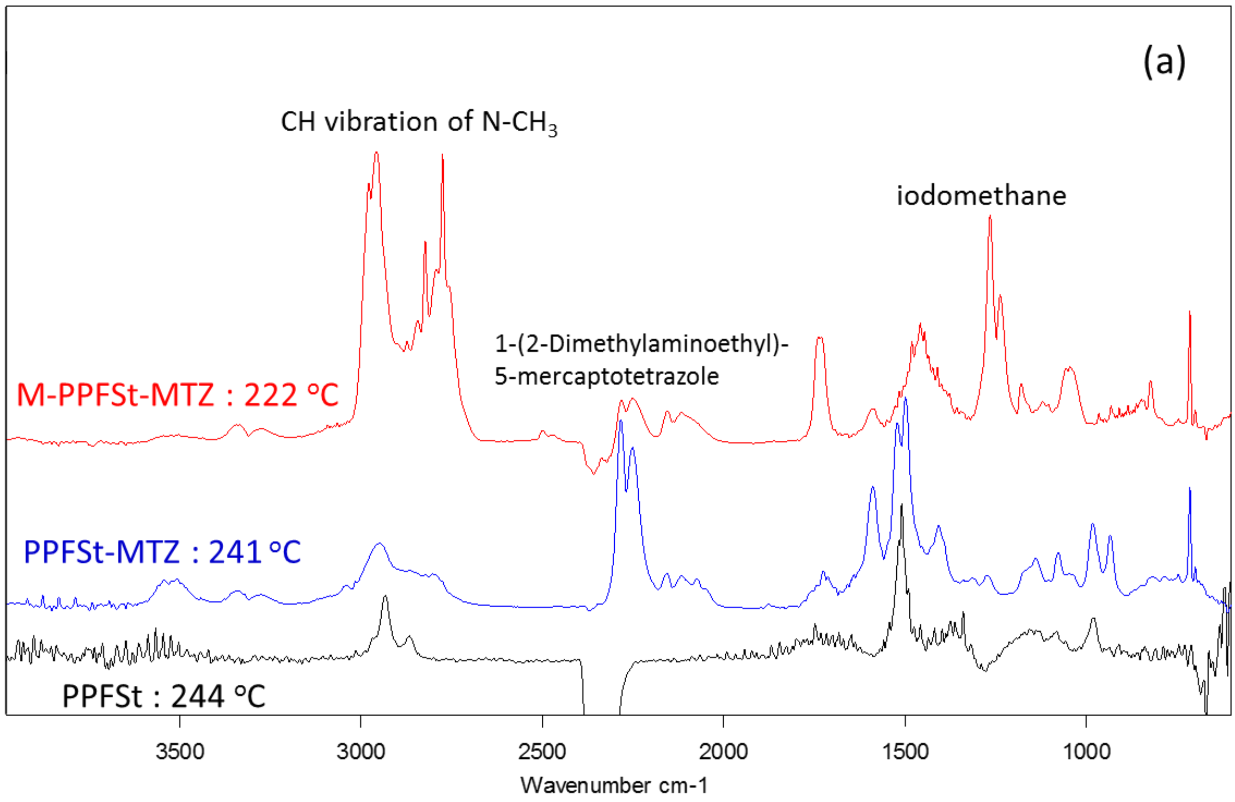

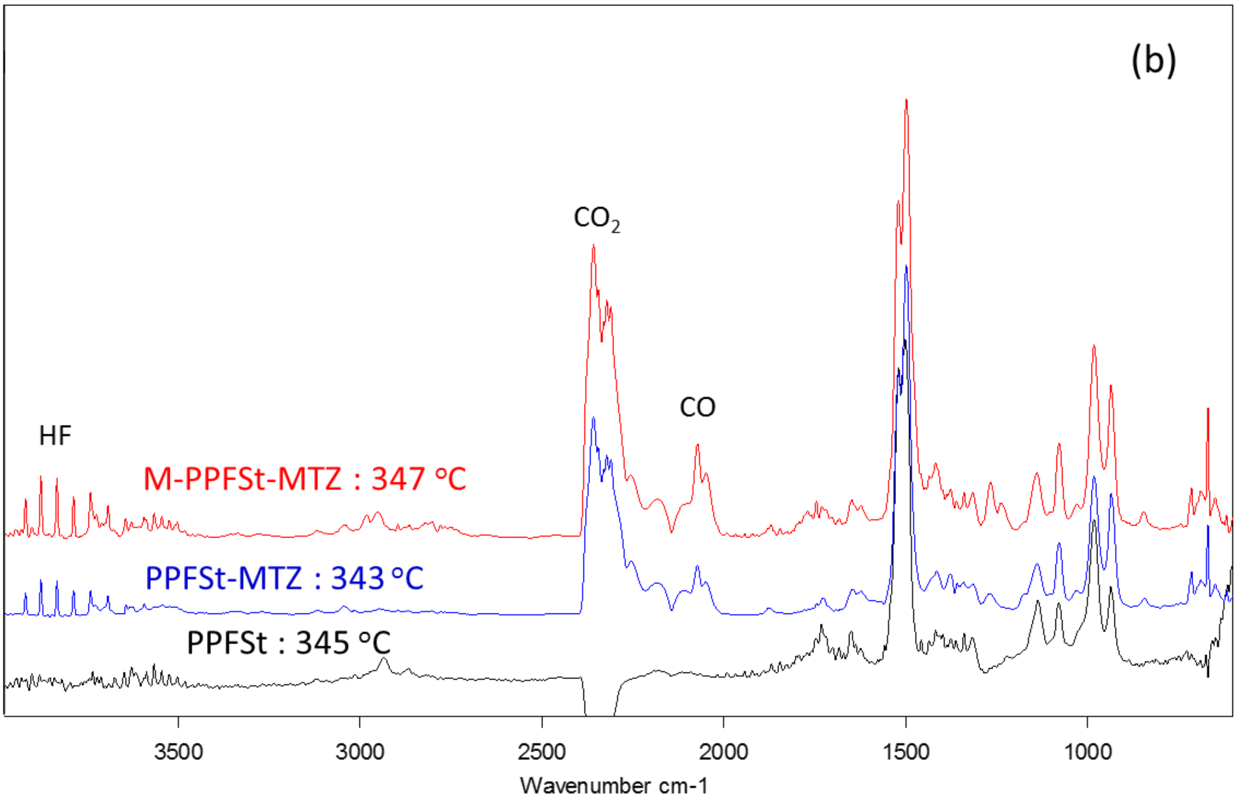


Figure S3. FT-IR spectrum of decomposed gases from first degradation step (a) and polymer main chain degradation (b).
